# Benthic-Pelagic Coupling: Effects on Nematode Communities along Southern European Continental Margins

**DOI:** 10.1371/journal.pone.0059954

**Published:** 2013-04-02

**Authors:** Ellen Pape, Daniel O. B. Jones, Elena Manini, Tania Nara Bezerra, Ann Vanreusel

**Affiliations:** 1 Marine Biology Research Group, Ghent University, Ghent, Belgium; 2 National Oceanography Centre, Southampton, United Kingdom; 3 Institute of Marine Science - National Research Council, Ancona, Italy; Leibniz Center for Tropical Marine Ecology, Germany

## Abstract

Along a west-to-east axis spanning the Galicia Bank region (Iberian margin) and the Mediterranean basin, a reduction in surface primary productivity and in seafloor flux of particulate organic carbon was mirrored in the *in situ* organic matter quantity and quality within the underlying deep-sea sediments at different water depths (1200, 1900 and 3000 m). Nematode standing stock (abundance and biomass) and genus and trophic composition were investigated to evaluate downward benthic-pelagic coupling. The longitudinal decline in seafloor particulate organic carbon flux was reflected by a reduction in benthic phytopigment concentrations and nematode standing stock. An exception was the station sampled at the Galicia Bank seamount, where despite the maximal particulate organic carbon flux estimate, we observed reduced pigment levels and nematode standing stock. The strong hydrodynamic forcing at this station was believed to be the main cause of the local decoupling between pelagic and benthic processes. Besides a longitudinal cline in nematode standing stock, we noticed a west-to-east gradient in nematode genus and feeding type composition (owing to an increasing importance of predatory/scavenging nematodes with longitude) governed by potential proxies for food availability (percentage of nitrogen, organic carbon, and total organic matter). Within-station variability in generic composition was elevated in sediments with lower phytopigment concentrations. Standing stock appeared to be regulated by sedimentation rates and benthic environmental variables, whereas genus composition covaried only with benthic environmental variables. The coupling between deep-sea nematode assemblages and surface water processes evidenced in the present study suggests that it is likely that climate change will affect the composition and function of deep-sea nematodes.

## Introduction

Apart from benthos dependent on chemosynthesis, deep-sea sediment communities ultimately depend for their dietary requirements on organic matter (OM) that is produced in the euphotic zone. The quantity that is produced varies among seasons and regions, and is determined by the physical properties and dynamics of the euphotic zone [1]. The particulate OM (POM) that is exported from the euphotic zone comprises phyto- and zoodetritus, in addition to bacteria, protozoans, fecal pellets (which mainly contain phytoplankton cells and gut bacteria) and inorganic compounds [2]. The processes through which POM is transferred to the deep-sea bottom are collectively termed “the biological pump”. During its descent through the water column the POM particles are progressively broken down, and only a limited fraction (1% on average) arrives at the deep-sea bed [1]. The fraction of exported POM that reaches the deep-sea sediments, or the efficiency of the biological pump, is determined by water depth, the sinking velocity (dependent on, amongst others, the degree of POM aggregation and the seawater mineral content) and the rate of decomposition (dependent on the pelagic food web structure and seawater temperature) of the POM [3]. In addition, laterally advected water masses may transport sinking POM away from its point of origin [4]–[6].

Various time-series studies documented an elevation in standing stock or metabolic activity, or both, of deep-sea benthic organisms in response to a phytodetritus pulse [7]–[10]. Empirical evidence for the coupling between the pelagic and the deep-sea benthic realm comes from feeding experiments, showing rapid uptake of added phytodetrital matter by all benthic size-classes, from prokaryotes to megafauna [11]–[13], including those living at abyssal depths [14], [15]. Some authors, however, failed to detect a response of (some of) the deep-sea benthic biota under study [16]–[18].

Meiofauna, a size-based invertebrate group dominated by nematodes, are a ubiquitous and dominant metazoan component of deep-sea sediments [19], [20]. Most proof for benthic-pelagic coupling stems from significant correlations between meiofaunal parameters and abiotic variables related to OM input. The magnitude of the flux of particulate organic carbon (POC) to the seabed was documented to have a positive effect on meiobenthic abundance [21], [22]. The concentration of chlorophyll-a (chl-a), and the sum of chl-a and its breakdown products (i.e. chloroplastic pigment equivalents or CPE) are commonly used to quantify the size of the fresh and total (fresh+degraded) phytodetrital pool, respectively, within the sediments [23], [24]. Sommer and Pfannkuche [21], Soltwedel [25], Neira et al. [26], Tselepides et al. [27], and Lampadariou et al. [28] all described a positive association between meiobenthic or nematode standing stock and pigment concentrations. However, Danovaro et al. [29] and Shimanaga et al. [30] did not observe a relationship between meiofaunal abundance and pigment concentrations. As a consequence, it has been argued that not food quantity, but food quality structures deep-sea benthic assemblages [29]. Other factors that were reported to correlate with deep-sea meiofaunal abundances or composition are granulometric characteristics [31] and sedimentary organic matter content [32], [33]. However, the bulk of the OM within deep-sea sediments is refractory and thus organic matter content represents a poor measure of food availability [34].

We selected several study areas in southern Europe positioned along a west-east axis, which are characterized by differential trophic and oceanographic conditions, namely the Galicia Bank (“GB”) region, and several basins within the western and eastern Mediterranean. The GB is a seamount located on the northwestern Iberian margin, which is marked by relatively high primary productivity (∼220 g C m^−2^ yr^−1^; [35]) owing to intense, wind-driven seasonal upwelling [36]. However, unlike the non-seamount stations in the GB region and the Mediterranean, the waters atop of the GB are hydrodynamically active [37], and as such bottom currents may interfere with OM deposition. Mediterranean deep-sea sediments represent a highly oligotrophic environment, because of the general nutrient depletion in surface waters combined with the high water temperature promoting the degradation of sinking OM [38]. Within the Mediterranean, there is a well-established trophic divergence between the more productive western and the less productive eastern basin [39]–[41]. This gradient is generated by the higher nutrient input in the western Mediterranean owing to river runoff and the inflow of Atlantic surface water, and the outflow of relatively nutrient-rich Levantine Intermediate Water through the Strait of Gibraltar (also known as the inverse estuarine circulation) [42]. Nonetheless, there appears to be substantial regional heterogeneity in surface productivity within both the western and the eastern Mediterranean basin owing to hydrological features and river runoff [43], [44].

The aim of this study was to determine how differences in oceanographic and productivity regimes between our study areas are reflected in nematode community characteristics at different bathyal and abyssal water depths (1200, 1900 and 3000 m). Measures of surface productivity, seafloor POC flux and *in situ* OM quality and quantity were used to verify and describe the longitudinal trophic gradient. We hypothesized that the west-east decline in primary production and sedimentation results in lower standing stock and a gradient in the generic and trophic structure of the nematode assemblages. In addition, we assessed the importance of both POC flux and benthic environmental characteristics for the distribution and structure of nematode communities.

## Materials and Methods

### Ethics Statement

No specific permits were required for the described field studies since the locations are not privately-owned or protected in any way and no endangered or protected species were involved.

### Study Area

**Figure 1:**
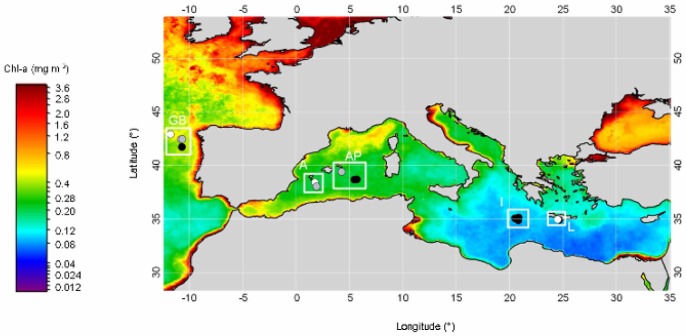
Average 2008 chlorophyll a levels of surface waters in the Mediterranean basin and the Galicia Bank region. These data were obtained from the Aqua MODIS satellite and were downloaded from Ocean Color Web**.** Symbols indicate locations of sediment samples taken at 1200 (white), 1900 (grey) and 3000 m (black) water depth. The white squares enclose samples from the same region. GB: Galicia Bank region, A: Algerian basin, AP: Algero-Provençal basin, I: Ionian basin, L: Levantine basin.

The Galicia Bank (“GB”) is a seamount situated on the Iberian margin, about 200 km off the Galician coast. It is separated from the shallower parts of the continental margin by the Galicia Interior basin, which has an approximate depth of 3000 m (Fig. 1). The dome-shaped GB seamount has a relatively flat quasi-rectangular summit (between ca. 620 and 900 m water depth) which is covered by a thick layer of foraminiferal ooze and is bounded by steep scarps [32], [37]. Duineveld et al. [37] measured high current velocities (5–30 cm s^−1^) at 1 m above the GB summit. We collected samples at (1200 m; GB1200) and southeast of the GB seamount (1900 and 3000 m; GB1900 and GB3000, respectively) (Fig. 1). Hence, GB1200 is a seamount station, whilst the deeper stations were positioned on the slope and the abyssal plain. The oceanographic area in which these stations were located is termed the GB region throughout the manuscript.

The Mediterranean Sea is split into the western and eastern Mediterranean basin by the Strait of Sicily. The western Mediterranean stations were situated in the Algerian (“A”, A1200 and A1900) and in the Algero-Provençal (“AP”, AP1900 and AP3000) basin. The eastern Mediterranean samples were collected in the Ionian Sea (“I”, I3000) and in the northern Levantine basin (“L”, L3000), offshore southern Crete. Sediment samples originating from each region were positioned on a west-to-east axis with an increase in longitude according to GB<A<AP< I<L (Fig. 1).

### Sampling Strategy

Sediment samples were gathered during various expeditions undertaken in the frame of the BIOFUN (“Biodiversity and ecosystem functioning in southern European deep-sea environments: from viruses to megafauna”) project (Table 1). We initially planned to sample only with a multicorer, because this gear produces the least disturbed sediment samples [45]. However, because of absence or malfunctioning of the multicorer, most of our samples comprised sub-samples from box cores taken with multicorer cores. Both Galeron et al. [46] and Mokievskii et al. [22] found no significant differences in meiobenthic abundances between multicorer and box corer samples, whereas Bett et al. [45] observed twice as much meiofauna in multicorer compared to box corer samples. Samples taken in the same basin or region and at the same water depth during the same expedition were considered replicates of the same station. Note that the number of replicate deployments varied among stations (1–9; Table 1). The surface area of the sampling cores used was not constant, and measured 78.54, 10.18, 70.88, 56.45 and 69.4 cm^2^ during the RV *Belgica*, *Urania*, *Pelagia* 2008, *Pelagia* 2009 and the *Sarmiento De Gamboa* expeditions, respectively. As a consequence, the total area sampled for nematode community analysis varied among stations (Table 1). All analyses were conducted on the top 0–1 cm of the sediment cores.

**Table 1:** Sampling details.

Basin	Station	Period	No of replicates			Depth (m)	Lat	Long	Gear	RV
			N (total area sampled, cm^2^)	OG	P					
GB	GB1200	Jun/08	3 (235.6)	3	0	1139–1141	42.9	−11.8	BC	*Belgica*
GB	GB1200	Oct/08	0	0	3	1155–1219	42.9	−11.8	BC	*Pelagia*
GB	GB1900	Oct/08	3 (212.64)	3	1+ (2)	1770–1896	42.4–42.5	−10.8–−10.7	BC	*Pelagia*
GB	GB3000	Oct/08	3 (212.64)	3	(3)	3066–3072	41.7	−10.7	BC	*Pelagia*
A	A1200	Jun/09	3 (208.20)	3	3	1211–1214	38.4	1.8	MC	*SDG*
A	A1900	Jun/09	2 (138.80)	2	0	2004, 2016	38.0	1.9	MC	*SDG*
AP	AP1900	Nov/09	3 (169.35)	3	3	1582	39.4	4.3	MC	*Pelagia*
AP	AP3000	Jun/09	3 (208.20)	3	3	2841–2846	38.7	5.5–5.7	MC	*SDG*
I	I3000	Jun/08	7 (71.26)	7	9	2770–2808	34.9–35.1	20.5–20.8	BC	*Urania*
L	L1200	Jun/08	3 (30.54)	3	(3)	983–1143	35.0	24.6	BC	*Urania*
L	L3000	Jun/08	1 (10.18)	1	(3)	2458, 2647	34.9	24.5, 24.6	BC	*Urania*

Indicated are: the region of origin (GB: Galicia Bank region, A: Algerian basin, AP: Algero-Provençal basin, I: Ionian basin, L: Levantine basin), the period samples were collected in (month/year), station code (indicating basin and approximate water depth), the number of replicate samples for nematode community analysis (N; the total area of sediment sampled is shown in parentheses), granulometry and organics (OG) and pigment analysis (P) whereby samples taken from the same deployment (i.e. pseudoreplicates) are indicated in parentheses, water depth range, average geographical position (latitude and longitude expressed in decimal degrees; where the coordinates of replicates differed more than 1°, a range is given), sampling gear (BC: box corer; MC: multicorer), research vessel (RV; SDG: *Sarmiento De Gamboa*).

### Analysis of Environmental Variables

Granulometric analysis was conducted using a Malvern Mastersizer hydro 2000 G. Sediment fractions were classified according to the Wentworth scale [47]. Following freeze-drying and homogenization, samples were acidified with 1% HCl. After acidification and drying, total organic carbon (TOC) and nitrogen (TN) content were measured using a Flash EA 1112+ MAs 200 elemental analyser (Thermo Interscience). Total organic matter (TOM) content was determined after combustion of the sediment samples at 550°C.

Chlorophyll-a (chl-a) and phaeopigment analyses were carried out according to Lorenzen and Jeffrey [48]. Pigments were extracted (12 h at 4°C in the dark) from triplicate superficial (0–1 cm) sediment samples (±1 g), using 5 ml of 90% acetone. Extracts were analysed fluorometrically to estimate chl-a, and after acidification with 200 ml 0.1 N HCl, to estimate phaeopigments. Chloroplastic pigment equivalents (CPE) constitute the sum of chl-a and phaeopigments. The ratio of chl-a and phaeopigments (chl-a:phaeo) was considered as a proxy for the “freshness” of the phytodetrital input.

Besides benthic environmental variables, we analyzed environmental data related to the pelagic realm. Net primary production (NPP) values were extracted from the Vertically Generalised Production Model (VGPM; resolution: 1°) described by Behrenfeld and Falkowski [49] and downloaded from http://www.science.oregonstate.edu/ocean.productivity/. The VGPM estimate of NPP values was based on satellite measurements of sea surface temperature (SST), surface water chl-a concentrations, and photosynthetically active radiation (PAR). Because only monthly data are available on NPP and in our study area variation in PAR and SST is negligible, we superimposed annual composite chl-a concentrations (Level-3 Aqua Modis data from 2008 with a resolution of 9 km; http://oceancolor.gsfc.nasa.gov) on a map displaying the locations of our samples to illustrate the heterogeneity in NPP. To this end we used the freely available HDF view, SAGA (System for Automated Geoscientific Analyses) and Quantum GIS (QGIS, v 1.7.4.) applications. Data on the particulate organic carbon (POC) flux to the seafloor were approximated on the basis of water depth and seasonal variation in NPP, calculated as the standard deviation divided by the mean of monthly NPP values, according to Lutz et al. [50].

NPP and POC flux to the seafloor were calculated for each sample location listed in Table 1. Owing to the 1° resolution of the input data for the VGPM model, replicate stations were often assigned equal NPP values and differences in seafloor POC flux were simply the result of the variability in measured water depth.

### Nematode Community Analysis

The sediment samples (0–1 cm sediment depth), fixed in seawater-buffered 4% formalin, were washed over a 32-µm mesh sieve and the meiofauna extracted from the sediment by Ludox centrifugation [51]. Meiofauna was then sorted, enumerated and identified at higher taxonomic level. Where possible, about 100 nematodes were hand-picked from each sample and identified to genus level. Since it was difficult to distinguish between *Microlaimus* and *Aponema*, specimens belonging to one of these genera were allocated to a *Microlaimus/Aponema* genus complex. Nematodes were grouped into four feeding types on the basis of the morphology of their buccal cavity *sensu* Wieser [52]: selective deposit feeders (1A), non-selective deposit feeders (1B), epistrate feeders and scavengers/predators (2B). Additionally, we measured length (L; µm) and maximal width (W; µm) for each nematode to estimate individual wet weight (WW) using Andrassy’s formula [53], adjusted for the specific gravity of marine nematodes (i.e. 1.13 g cm^−3^; µg WW ind^−1^ = L × W^2^/1500 000). Individual biomass (µg C ind^−1^) was then estimated as 12.4% of WW (Jensen 1984). Total nematode biomass (µg C 10 cm^−2^) in each sample was calculated as the product of nematode density (ind. 10 cm^−2^) and the arithmetic mean of individual biomass values.

### Data Analysis

Longitudinal and bathymetric monotonic trends in environmental (seafloor POC flux, phytopigments, MGS, mud, TOC, TOM, TN and C:N) and univariate nematode variables (density, individual and total biomass and relative abundance of feeding types) for a given depth or longitude were investigated by means of partial Spearman rank correlations. The strength and direction of longitudinal and bathymetric gradients were indicated by r_long|depth_ (correlation with longitude, given depth) and r_depth|long_ (correlation with depth, given longitude), respectively_._ Fourth-root transformed relative nematode genus abundances were subjected to distance-based linear modeling (DISTLM) to determine (1) whether spatial variation in genus composition was mostly owing to longitude or to water depth (shown by the marginal tests) and if (2) depth/longitude contributed to the explained variation, given longitude/depth (checked by the conditional tests). Genus composition data were visualized using non-metric multi-dimensional scaling (nMDS). To assess which genera described most of the longitudinal variation in community structure, we employed a BEST analysis using the fourth-root transformed genus abundances and the Bray-Curtis resemblance matrix based thereon, at each approximate depth (1200, 1900 or 3000 m). This type of analysis can be seen as a generalization of the SIMPER routine as it searches for a subset of genera that can account for the whole continuous pattern [54]. A SIMPER analysis was conducted to identify the nematode genera that discriminated most between the seamount and the non-seamount stations.

To determine the importance of the abiotic environment to nematode standing stock and composition, we conducted Spearman rank correlation and RELATE analysis (by means of Spearman rank correlations), respectively. Because pigment data were mostly obtained from different deployments than nematode and other environmental variables (Table 1), replicate environmental and nematode values were averaged per station. As a measure of within-station variability in nematode genus composition we used relative dispersion obtained through the MVDISP routine [54], which we subjected to correlation tests with all environmental variables.

Univariate correlation tests were executed in R v 2.15.0 [55]. Partial correlations were obtained with the R package ppcor [56]. All other analyses were done in PRIMER v6 with the PERMANOVA+ add-on [57], [58]. Because GB1200 was the only seamount station, it was omitted from the analysis of bathymetrical and longitudinal trends in benthic (environmental and nematode) parameters and the correlation test between environmental and nematode community descriptors. Consequently, the data on nematode community structure and benthic environmental variables at the seamount station were presented separately from those at the non-seamount stations. Data were expressed as means ± standard error (SE).

## Results

### Longitudinal and Bathymetric Trends in NPP and Seafloor POC Flux

**Figure 2:**
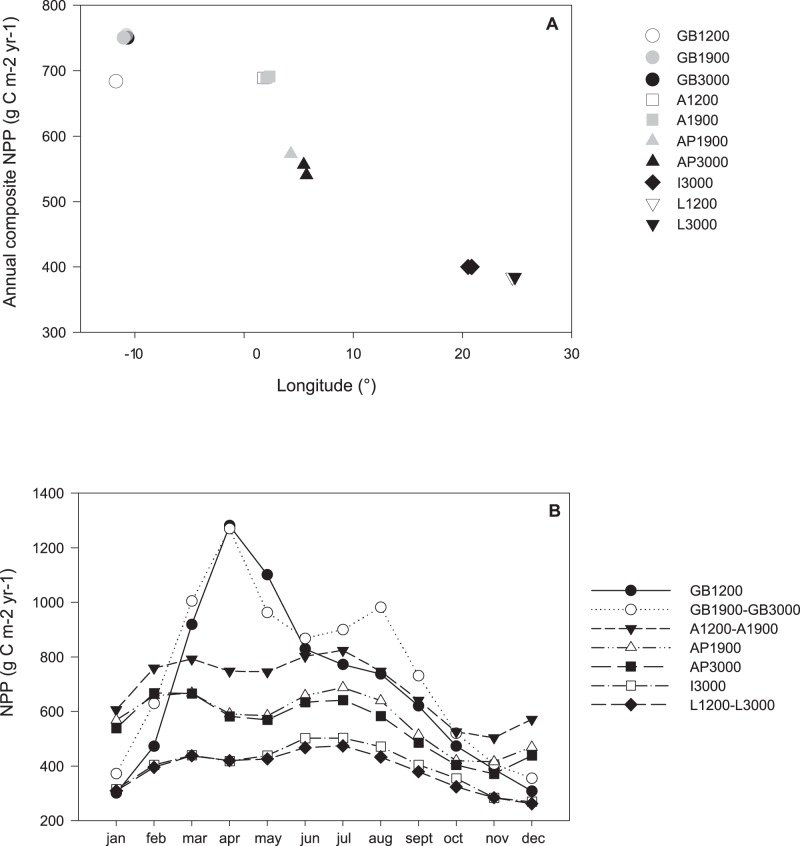
Satellite-based estimates of net primary productivity (NPP) in the Galicia Bank region and the Mediterranean. (A) Annual composite NPP in function of longitude. Symbols represent stations, indicating basin (shape) and water depth (colour). GB: Galicia Bank region, A: Algerian basin, AP: Algero-Provençal basin, I: Ionian basin, L: Levantine basin. (B) Monthly variation in NPP. Each line represents NPP values for stations located less than 1° longitude apart.

There was a significant reduction in NPP along the west-to-east axis from the GB region to the eastern Mediterranean basin (Fig. 2A; Spearman rank: r = -0.89, P<0.001). Because they were distanced by 1° longitude or more, some samples from the same basin, either from equal (i.e. the 3000 m samples from the Algero-Provençal and Ionian basin) or different water depths (1200 and 1900+3000 m samples from the GB region and 1900 and 3000 m samples in the Algero-Provençal basin), displayed differential NPP (Fig. 2). Nevertheless, on average, NPP ranged between 716.2 g C m^−2^ yr^−1^ at the GB region and 384.2 g C m^−2^ yr^−1^ in the Levantine Basin. Within the western Mediterranean, the Algerian basin (688 g C m^−2^ yr^−1^) exhibited elevated NPP relative to the Algero-Provençal basin (540.3–572.5 g C m^−2^ yr^−1^). In the eastern Mediterranean basin, there was a small drop in NPP between the Ionian (400.0 g C m^−2^ yr^−1^) and the Levantine Sea (384.2 g C m^−2^ yr^−1^).

Seasonal variability in NPP at the GB region (0.43) was more than twice that in the Mediterranean, where values slightly increased from west to east with 0.16, 0.18, 0.20 and 0.19 in the Algerian, Algero-Provençal, Ionian and Levantine basin, respectively (see also Fig 2B). The GB1200 station experienced maximal NPP in April, while the deeper stations in the GB region showed an additional, but less pronounced NPP peak in August (Fig. 2B). The Mediterranean stations experienced two NPP maxima per year; one in March and another one in July.

**Figure 3:**
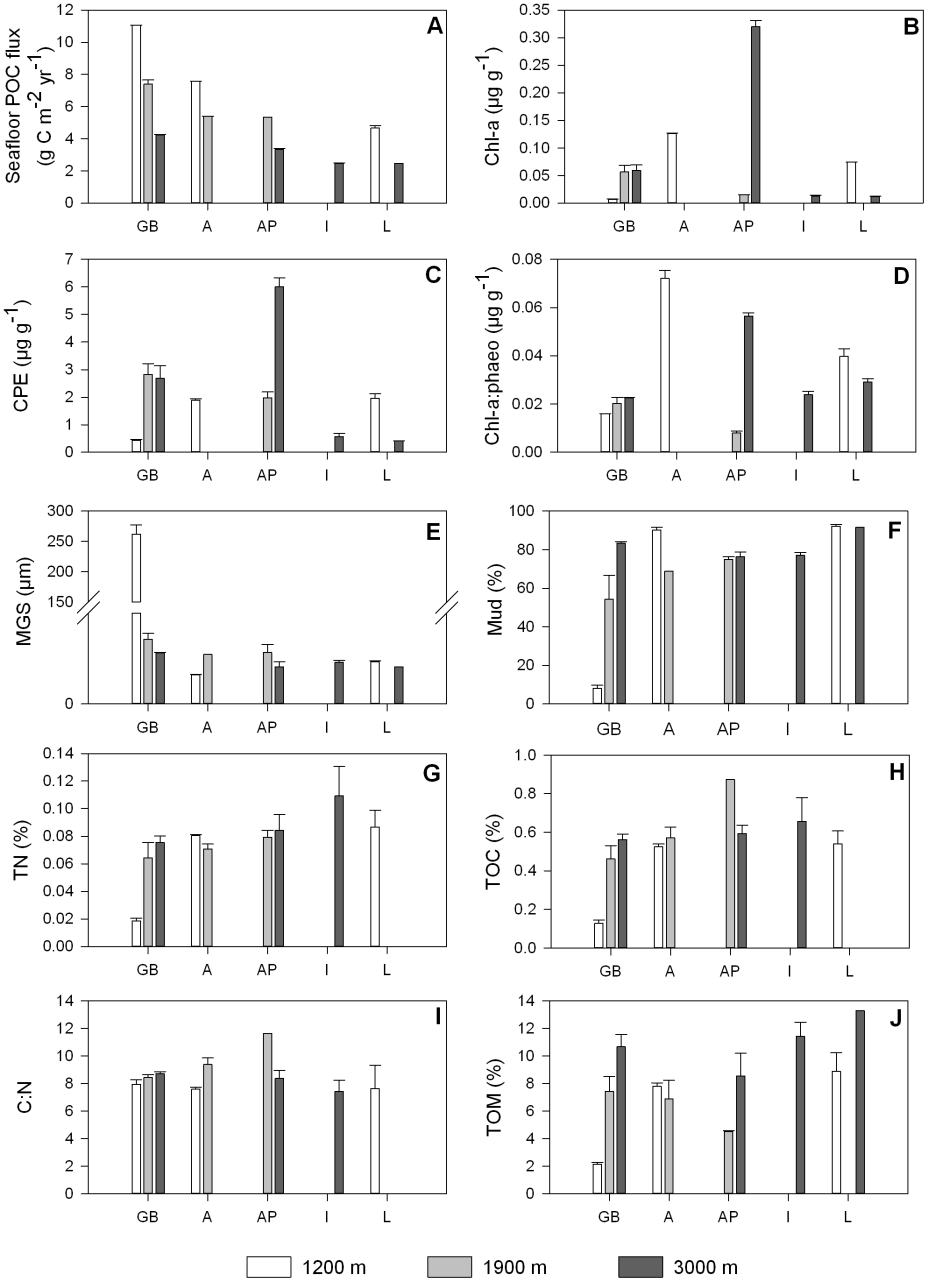
Longitudinal and bathymetric trends in environmental variables. The basins sampled are displayed on the x-axis (GB: Galicia Bank region, A: Algerian basin, AP: Algero-Provençal basin, I: Ionian basin, L: Levantine basin) and are ordered from west to east. The colour of the bars indicates approximate water depth (1200 m: white, 1900 m: grey, 3000 m: dark grey). Bars represent average values; error bars denote standard errors. POC flux: annual particulate organic carbon flux to the seafloor, chl-a: chlorophyll a, CPE: chloroplastic pigment equivalents, chl-a:phaeo: chlorophyll a: phaeopigments ratio, MGS: median grain size, TOM: % of total organic matter, TOC: % of total organic carbon, TN: % of total nitrogen, C:N : molar carbon:nitrogen ratio.

For a given water depth, seafloor POC flux related negatively with longitude (Fig. 3A; Table 2). When longitude was fixed, POC flux declined along the bathymetrical axis.

**Table 2:** Partial Spearman rank correlations with longitude, given depth (r_long|depth_), and depth, given longitude (r_depth|long_) for environmental and nematode variables.

		r_long|depth_	r_depth|long_
**POC flux**		−0.94***	−0.93***
**Chl-a**	*incl. AP3000*	−0.54***	−0.24
	*excl. AP3000*	−0.71***	−0.69*
**CPE**	*incl. AP3000*	−0.64***	−0.07
	*excl. AP3000*	−0.77***	−0.53**
**Chla:phaeo**	*incl. AP3000*	0.20	−0.13
	*excl. AP3000*	0.31	−0.35
**MGS**		−0.55***	−0.13
**Mud**		0.49**	−0.003
**TOM**		0.42*	0.51**
**TOC**		0.21	0.14
**TN**		0.25	0.02
**C:N**		−0.19	0.09
**Density**		−0.67***	−0.57***
**Total biomass**		−0.75***	−0.38*
**Ind. biomass**		−0.65***	0.09
**1A**		−0.23	0.05
**1B**		0.02	0.16
**2A**		−0.32	−0.22
**2B**		0.67***	0.10

Environmental variables: POC flux (annual particulate organic carbon flux to the seafloor), chl-a (chlorophyll a), CPE (chloroplastic pigment equivalents), chl-a:phaeo (chlorophyll a: phaeopigments ratio), MGS (median grain size), mud, TOM (% of total organic matter), TOC (% of total organic carbon), TN (% of total nitrogen), C:N (molar carbon: nitrogen ratio). Nematode variables: density, total and ind. biomass, and relative abundances of feeding types 1A (selective deposit feeders), 1B (non-selective deposit feeders), 2A (epistrate feeders) and 2B (predators/scavengers). Station AP3000 represented an outlier for the pigment data and analysis was conducted with and without this station. All analyses, except for POC flux, were run without seamount station GB1200. The number of asterisks denotes the statistical significance level with *P≤0.05, **0.05<P≤0.01, and ***0.01<P≤0.001.

### Benthic Environmental and Nematode Community Characteristics of the Seamount Station

**Figure 4:**
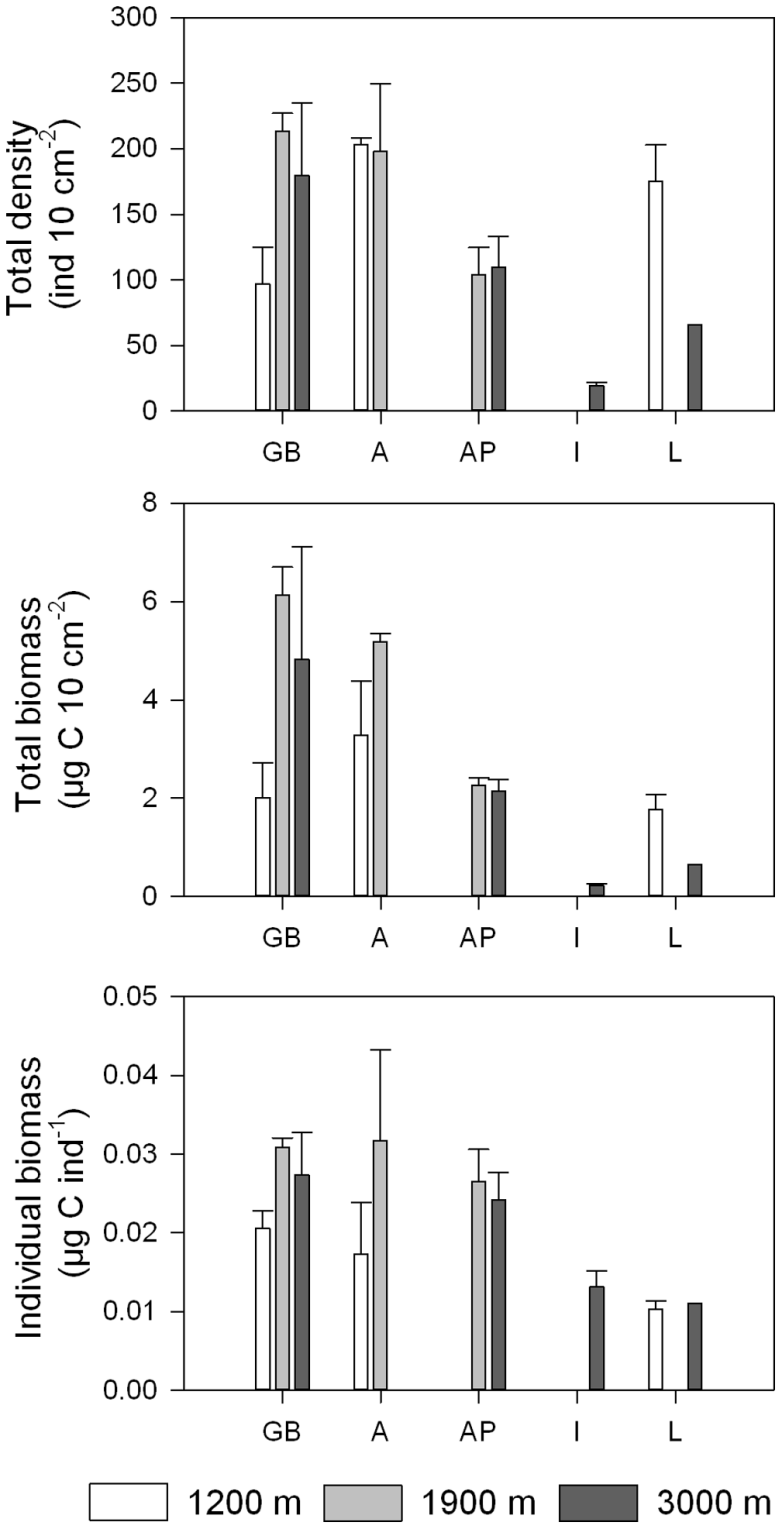
Longitudinal and bathymetric trends in densities, total and individual biomass of nematodes. The basins sampled are displayed on the x-axis (GB: Galicia Bank region, A: Algerian basin, AP: Algero-Provençal basin, I: Ionian basin, L: Levantine basin) and are ordered from west to east. The colour of the bars indicates approximate water depth (1200 m: white, 1900 m: grey, 3000 m: dark grey). Bars represent average values; error bars denote standard errors.

**Figure 5:**
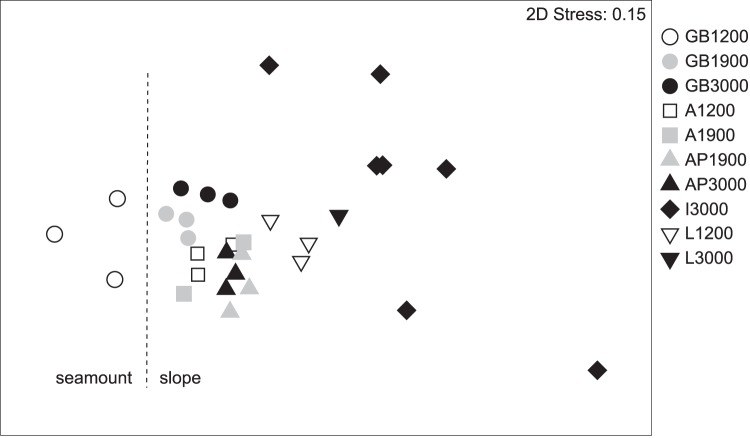
Non-metric multi-dimensional scaling (nMDS) plot of fourth-root transformed relative nematode genus abundances per station. The dashed line separates the seamount station from the non-seamount stations. Station codes are explained in Table 1.

**Figure 6:**
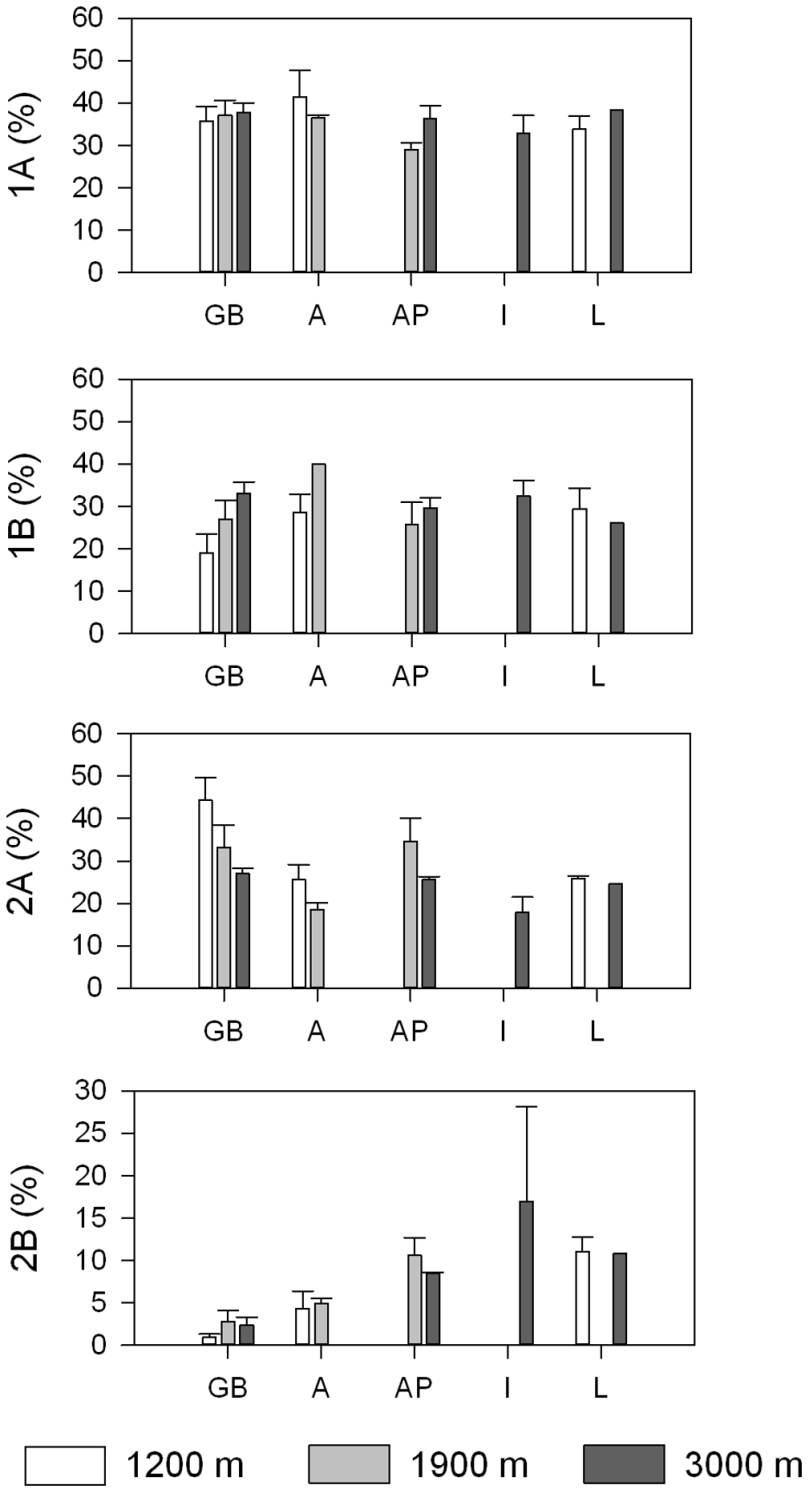
Longitudinal and bathymetrical trends in the relative abundance of nematode feeding types. The basins sampled are displayed on the x-axis (GB: Galicia Bank region, A: Algerian basin, AP: Algero-Provençal basin, I: Ionian basin, L: Levantine basin) and are ordered from west to east. The colour of the bars indicates approximate water depth (1200 m: white, 1900 m: grey, 3000 m: dark grey). Bars represent average values; error bars denote standard errors. 1A: selective deposit feeder, 1B: non-selective deposit feeder, 2A: epistrate feeder, 2B: predator/scavenger).

Even though we obtained the highest seafloor POC flux values for seamount station GB1200 (Fig. 3A), this station was characterized by the lowest phytopigment concentrations of all (chl-a: 0.0070±0.0006 µg g^−1^, CPE: 0.441±0.035 µg g^−1^) (Fig. 3B–C). In addition, we observed divergently low values for TN (0.045±0.002%; Fig. 3G), TOC (0.13±0.02%; Fig. 3H) and TOM (2.13±0.14%; Fig. 3J). The samples collected at GB1200 contained low numbers of nematodes (96.9±27.7 ind. 10 cm^−2^) relative to the 1200 m station in the western Mediterranean (203.1±5.0 ind. 10 cm^−2^) and the deeper stations in the GB region (GB1900∶213.5±13.7 ind. 10 cm^−2^; GB3000∶180.0±54.7 ind. 10 cm^−2^) (Fig. 4). Fig. 5 shows that the nematode generic composition at the seamount station diverged from that at the non-seamount stations. The SIMPER analysis indicated that this divergence (average dissimilarity: 60.4%) was partly driven by the higher relative abundance of *Bolbolaimus*, *Desmodora*, *Metadesmolaimus, and Microlaimus/Aponema* and the absence of *Sphaerolaimus* at GB1200 (Table 3). *Bolbolaimus* was one of the 13 genera that were restricted to station GB1200. Similar to the non-seamount stations, the seamount station was dominated by deposit-feeding nematodes (feeding types 1A +1B; 54.9±5.3%). Of all stations, GB1200 had the highest fraction of epistrate feeders (2A; 44.2±5.5%) and the lowest fraction of predators/scavengers (2B; 0.88±0.45%; Fig. 6).

**Table 3:** Average (SE) relative abundances (%) of dominant nematode genera (≥3%) per station.

GB1200		GB1900		GB3000		A1200			A1900	
*Microlaimus/Aponema*	12.4 (3.6)	*Acantholaimus*	15.7 (1.5)	*Theristus*	9.6 (1.5)	*Amphimonhystrella*		8.3 (1.6)	*Monhystrella*	11.4 (1.9)
*Leptolaimus*	11.3 (1.4)	*Daptonema*	14.6 (2.3)	*Acantholaimus*	9.5 (1.5)	*Daptonema*		7.4 (3.3)	*Halalaimus*	7.6 (0.1)
*Desmodora*	10.3 (4.3)	*Microlaimus/Aponema*	7.9 (3.2)	*Microlaimus/Aponema*	9.5 (1.1)	*Halalaimus*		7.3 (1.0)	*Acantholaimus*	7.3 (4.4)
*Bolbolaimus*	8.1 (3.5)	*Diplopeltula*	5.0 (2.6)	*Daptonema*	9.1 (1.2)	*Neochromadora*		6.8 (0.9)	*Amphimonhystrella*	7.1 (0.5)
*Richtersia*	4.2 (2.1)	*Desmoscolex*	4.7 (0.5)	*Tricoma*	7.4 (3.4)	*Acantholaimus*		6.8 (1.2)	*Richtersia*	6.7 (0.9)
*Metadesmolaimus*	4.0 (1.3)	*Halalaimus*	4.1 (1.3)	*Halalaimus*	6.6 (0.9)	*Leptolaimus*		5.9 (2.7)	*Sabatieria*	6.4 (3.1)
*Pselionema*	4.0 (1.3)	*Thalassomonhystera*	4.0 (2.1)	*Monhystrella*	6.1 (0.3)	*Pselionema*		4.8 (1.2)	*Daptonema*	5.3 (0.5)
*Acantholaimus*	3.8 (1.7)	*Leptolaimus*	3.9 (2.7)	*Diplopeltula*	4.0 (0.8)	*Richtersia*		4.2 (2.3)	*Prototricoma*	4.0 (0.7)
*Halalaimus*	3.8 (0.8)	*Monhystrella*	3.2 (1.2)	*Thalassomonhystera*	4.0 (1.4)	*Desmoscolex*		4.1 (1.2)	*Leptolaimus*	3.7 (2.0)
*Daptonema*	3.7 (0.5)			*Desmoscolex*	4.0 (0.1)	*Tricoma*		3.7 (2.5)	*Molgolaimus*	3.5 (0.7)
				*Prototricoma*	3.1 (0.9)	*Monhystrella*		3.5 (0.7)	*Diplopeltula*	3.2 (0.7)
**AP1900**		**AP3000**		**I3000**		**L1200**			**L3000**	
*Acantholaimus*	13.5 (2.5)	*Acantholaimus*	13.4 (2.1)	*Monhystrella*	13.2 (5.1)	*Acantholaimus*	10.7 (1.8)		*Acantholaimus*	16.9
*Halalaimus*	13.5 (0.9)	*Halalaimus*	12.0 (1.6)	*Acantholaimus*	12.7 (3.6)	*Theristus*	5.8 (2.5)		*Halalaimus*	15.4
*Neochromadora*	5.4 (2.4)	*Amphimonhystrella*	6.7 (0.2)	*Sphaerolaimus*	7.5 (2.9)	*Pselionema*		5.6 (1.8)	*Thalassomonhystera*	10.8
*Microlaimus/Aponema*	5.4 (2.8)	*Daptonema*	6.6 (2.7)	*Halalaimus*	5.2 (1.5)	*Halalaimus*		5.0 (0.8)	*Metasphaerolaimus*	7.7
*Daptonema*	4.7 (2.9)	*Monhystrella*	4.4 (0.9)	*Molgolaimus*	5.1 (2.2)	*Richtersia*		4.7 (0.6)	*Enchonema*	4.6
*Monhystrella*	4.7 (0.2)	*Leptolaimus*	3.5 (0.9)	*Theristus*	5.0 (2.6)	*Sabatieria*		4.7 (1.0)	*Marylinnia*	4.6
*Amphimonhystrella*	3.8 (1.5)	*Aegialoalaimus*	3.2 (0.7)	*Thalassomonhystera*	4.8 (2.4)	*Molgolaimus*		4.1 (0.3)	*Oxystomina*	4.6
*Metasphaerolaimus*	3.8 (2.1)			*Metasphaerolaimus*	4.6 (2.5)	*Monhystrella*		3.8 (0.1)	*Pselionema*	4.6
				*Aegialoalaimus*	3.9 (1.9)	*Marylinnia*		3.8 (0.5)	*Diplopeltula*	3.1
				*Sabatieria*	3.6 (2.0)	*Prototricoma*		3.8 (0.5)	*Manganonema*	3.1
				*Diplopeltula*	3.1 (1.7)	*Sphaerolaimus*		3.7 (1.6)	*Sphaerolaimus*	3.1
						*Desmoscolex*		3.2 (0.8)		

The number of replicate samples per station is indicated in Table 1.

### Benthic Environmental and Nematode Community Characteristics of the Non-seamount Stations

#### Longitudinal and bathymetric trends in benthic environmental variables

The partial Spearman rank correlation coefficients describing longitudinal and bathymetrical trends at a fixed depth and longitude, respectively, are presented in Table 2. Station AP3000 displayed relatively low POC deposition, albeit elevated sedimentary phytopigment levels (chl-a: 0.3205±0.0108 µg g^−1^, CPE: 6.005±0.324 µg g^−1^) in comparison with the stations in the GB region and the Algerian basin, and with AP1900. We found a significant longitudinal decline in chl-a and CPE levels (Fig. 3B–C), which was more pronounced (i.e. more negative value of r_long|depth_) when station AP3000 was excluded from the analysis. Mud content (Fig. 3F) and MGS (Fig. 3E) increased and decreased, respectively, from west to east. The percentage of TOM showed a positive relation with longitude and with water depth (Fig. 3J). Sedimentary TOC (Fig. 3H), TN (Fig. 3G) and consequently C:N (Fig. 3I) values did not change with depth or longitude.

### Longitudinal and Bathymetric Trends in Nematode Community Characteristics

The relative densities of the various meiofaunal taxa encountered at each (seamount+non-seamount) station are listed in Table S1. Nematodes prevailed at all stations, accounting for 70.0–96.1% of meiofaunal abundance. The second most numerous taxon were the copepods (adults+nauplii; 1.8–25.0%). At some stations polychaetes, tardigrades or rotifers represented more than 1% of total meiofaunal abundance.

Table 2 contains the coefficients of the Spearman rank correlations for nematode density and biomass with longitude and depth. Nematode standing stock (i.e. total densities and biomass) declined with longitude and with depth. Individual nematode biomass also decreased from west to east but remained constant with increasing water depth.

For all stations (seamount and non-seamount), we recorded 150 nematode genera of which the numerically dominant ones (contributing ≥3% of total abundance) are listed in Table 3. *Acantholaimus* and *Halalaimus* were amongst the dominant genera at every station. Only eleven genera were encountered at all stations. When water depth was fitted first in the DISTLM model (excluding GB1200), there was a graded transition in nematode genus composition from west to east (sequential tests, depth: P<0.05; longitude: P<0.001; Fig. 5). Additionally, when longitude was fixed, depth contributed significantly to the explained variation in genus composition (sequential DISTLM tests, longitude: P<0.001; depth: P<0.05). Longitude (depth fitted first: 18.6%, longitude fitted first: 18.5%) explained a greater fraction of the variability in nematode genus composition than water depth (depth fitted first: 6.9%, longitude fitted first: 7.0%). The BEST analysis showed that at 1200 m depth the genus *Chromadorina* was most responsible (R = 0.99) for the divergence between A1200 and L1200, which was absent from the latter station. At the 1900 m stations, *Manganonema* and *Spirodesma*, both only found at the GB region, were steering the longitudinal gradient in genus composition (R = 0.62). *Linhomoeus* (absent from the Ionian and Levantine basin), *Metasphaerolaimus* (more prevalent in the Levantine and Ioanian basin) and *Gnomoxyala* (restricted to the Ionian Sea) were the genera mainly responsible for the cline in genus composition at the abyssal stations (R = 0.75).

The trophic structure of the nematode communities at all (seamount+non-seamount) stations is illustrated in Fig. 6. Deposit feeders (1A +1B) prevailed at all stations and accounted for 45.7 to 85.7% of total nematode abundance. Except for half of the I3000 samples, in which epistrate feeders (2A) attained lowest relative abundances, predators/scavengers (2B) were represented the least (range: 0–15.4%). The relative abundance of predatory/scavenging nematodes displayed a positive longitudinal gradient (Table 2).

### Correlations between Seafloor POC Flux and Benthic Environmental Variables

Seafloor POC flux showed an inverse relationship with station-averaged TOM (Spearman rank, r = −0.78, P = 0.01).

### Environmental Drivers of Nematode Assemblages

**Figure 7:**
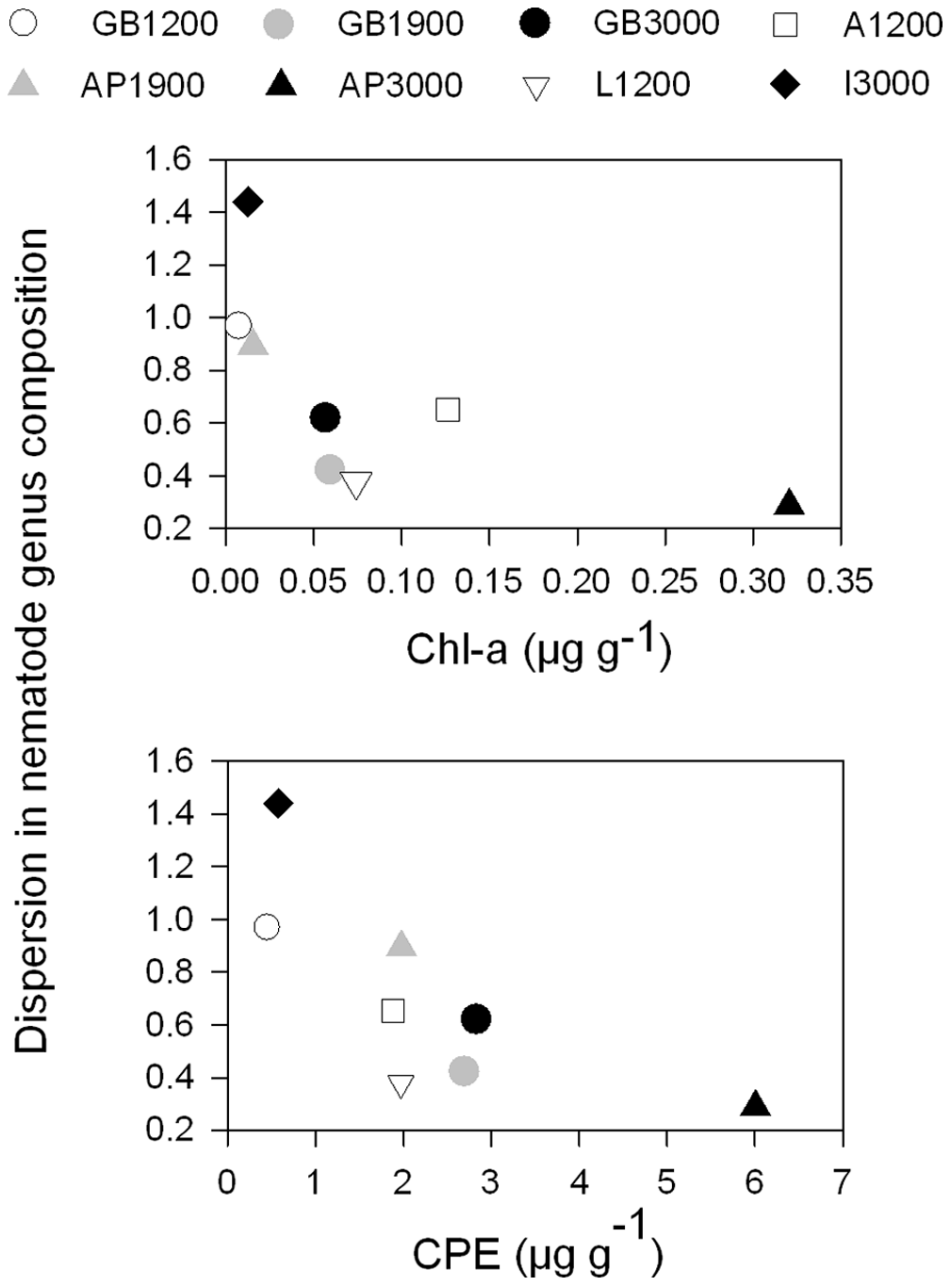
Within-station variability in nematode genus composition in function of sedimentary phytopigment concentrations. Top: chlorophyll a (chl-a) concentrations. Bottom: chloroplastic pigment equivalents (CPE). Pigment data were missing for station A1900. Only one sample was collected at L3000, prohibiting the assessment of within-station variability in community structure.

The Spearman rank correlations computed between environmental variables and nematode community descriptors are shown in Table 4. Nematode density and biomass were both correlated with the magnitude of seafloor POC flux, but related more strongly to benthic parameters like TOC (in the case of nematode density) and TN (total biomass). Nematode individual biomass was impacted positively by sedimentary C:N levels, and displayed an inverse relationship with sedimentary mud and TN content. The generic structure of the nematodes related significantly to sedimentary organic matter and nitrogen concentrations. Although chlorophyll pigments were not correlated with nematode genus composition, the variability between replicate samples of the same station declined with increasing pigment concentration (Spearman rank, chl-a: r = -0.83, P<0.05; CPE: r = -0.76, P<0.05; Fig. 7). After omission of station I3000, from which an aberrantly high number of replicates (n = 7) were collected, this relationship was still significant for chl-a (Spearman rank, r = −0.79, P = 0.05), but not for CPE (r = −0.68, P = 0.11).

**Table 4:** Spearman rank correlations between station-level nematode and environmental variables.

	POC flux	Chl-a	CPE	Chl-a:phaeo	TOM	TOC	TN	C:N	MGS	mud
**Density**	0.85**	0.62	0.60	0.12	−0.52	−0.88**	−0.71	0.12	0.28	−0.32
**Ind. biomass**	0.50	0.14	0.69	−0.55	−0.65	−0.10	−0.93**	0.76*	0.63	−0.85**
**Total biomass**	0.77*	0.40	0.62	−0.26	−0.67	−0.52	−0.98***	0.57	0.57	−0.62
**Genus comp.**	0.02	−0.17	0.30	−0.23	0.44*	0.40*	0.40*	0.04	−0.12	−0.14
**1A**	0.20	0.17	−0.14	0.45	0.25	−0.76*	−0.48	−0.10	−0.40	0.27
**1B**	−0.08	0.24	0.31	0.14	0.15	0.14	0.05	−0.05	−0.02	−0.08
**2A**	0.35	0.19	0.64	−0.60	−0.45	−0.12	−0.45	0.60	0.65	−0.22
**2B**	−0.57	−0.38	−0.64	0.17	0.37	0.60	0.79*	−0.36	−0.33	0.38

Environmental variables: POC flux (annual particulate organic carbon flux to the seafloor), chl-a (chlorophyll a), CPE (chloroplastic pigment equivalents), chl-a:phaeo (chlorophyll a: phaeopigments ratio), TOM (% of total organic matter), TOC (% of total organic carbon), TN (% of total nitrogen), C:N (molar carbon: nitrogen ratio) MGS (median grain size) and mud. Nematode variables: density, total and ind. biomass, and relative abundances of feeding types 1A (selective deposit feeders), 1B (non-selective deposit feeders), 2A (epistrate feeders) and 2B (predators/scavengers). Correlations between genus composition (genus comp.) and environmental variables were obtained through the RELATE procedure. The number of asterisks denotes the statistical significance level with *P≤0.05, **0.05<P≤0.01, and ***0.01<P≤0.001.

We found only few significant associations between the relative densities of nematode feeding types and environmental variables at the station level (Table 4). Selective deposit feeders (feeding type 1A) were affected negatively by sedimentary TOC levels, whereas predatory/scavenging nematodes (2B) contributed more to total nematode abundances in sediments with elevated TN levels.

## Discussion

### Longitudinal Trend in NPP

Our Vertically Generalized Production Modeled NPP data revealed a decline in surface productivity along a longitudinal transect from the GB region to the eastern Mediterranean, in agreement with previously reported *in situ* measurements [59] and other satellite-based estimates [41], [44] for the Mediterranean Sea. The longitudinal drop in productivity was more pronounced for the *in situ* measurements of Moutin and Raimbault [59] (factor 2.2–2.9) compared to our data (1.8) and those of Bosc et al. [44] (1.5). Standard algorithms used to convert ocean colour data to chlorophyll concentrations have been proven to systematically overestimate actual concentrations in the Mediterranean, especially in the oligotrophic parts of the basin [44], [60]. However, the west-east difference in productivity reported in this study, in which standard algorithms were used, is larger than that recorded by Bosc et al. [44] who employed a bio-optical algorithm adapted for Mediterranean waters.

The NPP data estimated here were high compared to those reported previously for the same oceanographic regions. This may be related to the usage of different algorithms, which may provide divergent estimates of NPP [61], [62]. Joint el al. [35] provided a regional NPP estimate for the northwestern Iberian margin, where the GB is situated, of ∼220 g C m^−2^ yr^−1^, which is about three times lower than our value for the GB1200 station (693.5 g C m^−2^ yr^−1^). This may point to a local NPP maximum associated with the GB, which has been observed before for some seamounts [63]. However, unlike seamount station GB1200, the deeper stations in the GB region experience, besides a chlorophyll maximum in spring, a NPP peak in late summer, resulting in higher annual NPP. This second NPP maximum may result from offshore transport of phytoplankton produced at the shelf following coastal upwelling during summer [35]. Also for the various Mediterranean regions, our NPP approximations (Fig. 2A) were consistently a factor 3 to 4 higher than the satellite-based values of Bosc et al. [44] (Algerian basin: 162.5 g C m^−2^ yr^−1^, Algero-Provençal basin: 153 g C m^−2^ yr^−1^, Ionian basin: 120.4 g C m^−2^ yr^−1^, Levantine basin: 106.3 g C m^−2^ yr^−1^). Because of the presence of Saharan dust in Mediterranean surface waters, standard algorithms, such as VGPM, tend to overestimate NPP in this basin [44], [60]. Bosc et al. [44] employed a regional algorithm, adapted for Mediterranean waters, which was believed to generate less biased NPP values.

### Longitudinal Trend in Seafloor POC Flux

The productivity gradient between the GB region and the eastern Mediterranean was accompanied by a reduction in seafloor POC flux, conforming with sediment trap data for the Mediterranean basin [39]. However, several factors may have resulted in a bias in these estimated POC flux data.

The POC flux calculated for the seamount station was most probably an overestimate since local strong near-bottom currents may have diverted the POC rain [4]–[5], [64]. The coarse sediment texture at the GB was previously ascribed to the winnowing of the fine sediment fraction by strong bottom currents [37], [65]. At station GB1200, maximal estimated POC deposition was associated with minimal benthic pigment concentrations, pointing to a decoupling between NPP and POC deposition (but see later). However, the POC flux we approximated for GB1200 (Fig. 2A) was similar to the values of Duineveld et al. [37] at 800 m depth (6.2–12.8 g C m^−2^ yr^−1^).

The POC sedimentation rates estimated for the Mediterranean stations were either lower than or comparable to those previously reported. At 3000 m depth in the Algero-Provençal basin, Zúñiga et al. [6] recorded a POC flux of 1 g C m^−2^ yr^−1^, which is one third of the value we found for station AP3000. In the Antikythira strait, which connects the Aegean with the Ionian Sea, the amount of POC collected with a sediment trap placed 1345 m deep was 3.9 g C m^−2^ yr^−1^ [66], which is of the same order as the POC flux we estimated for the same depth in the Levantine Sea (4.7±0.1 g C m^−2^ yr^−1^). In the Cretan Sea, north of Crete, Danovaro et al. [39] measured a four times lower POC flux of 1.2 g C m^−2^ yr^−1^, inferred from sediment trap data. The divergence between modeled POC flux and that measured by sediment traps may be related to the systematic bias associated with the latter caused by, amongst others, inclusion of swimmers, hydrodynamic biases, degradation of trapped organic material [67].

Additional potential bias in the POC flux estimated via the algorithm of Lutz et al. [50] may be introduced by (1) the limited coverage of the data, on which the algorithm was based, relative to the World ocean surface (including only two (rather productive) locations from the Mediterranean), and (2) the fact that the seasonal variability in NPP is not the only factor that governs the amount of POC that is transported to depth. First of all, POC that sinks from the euphotic zone does not only comprise phytoplankton-derived material, but also consists of fecal pellets and zooplankton moults [2], [3]. The contribution of fecal pellets to the POC rain can be substantial (up to 100% of total POC flux) and varies between regions [68]. Secondly, the composition and activity (which is affected by seawater temperature) of pelagic zooplankton and bacterial communities, as well as the overall food-web structure determines the efficiency of the biological pump [69]. Siokou-Frangou et al. [70] describe the disparity in the composition of plankton communities between the western and the eastern Mediterranean, which may lead to differences in POC transport efficiency. The inclusion of data on circulation patterns, and plankton community and food web structure may lead to a refinement of the algorithm to estimate POC flux.

### Benthic-pelagic Coupling: Effects on the Seafloor Environment

The longitudinal cline in surface productivity and resulting sedimentation rates between the GB region and the Levantine basin was mirrored in the benthic phytopigment concentrations, consistent with Pusceddu et al. [71] (Portugese-Cretan margin) and Gambi and Danovaro [72] (western versus eastern Mediterranean).

At seamount station GB1200 (high POC flux, low pigment concentrations) and abyssal plain station AP3000 (low POC flux, high pigment concentrations), seafloor POC flux data did not appear to match with sedimentary pigment levels. We argue that the high pigment levels observed for AP3000 relative to the GB stations and A1200 are primarily caused by the different timing of the expeditions during which these stations were sampled. The pigment samples from AP3000 were collected in June, when NPP values were nearly maximal (Fig. 2B). In contrast, sampling at the GB region and at station AP1900 was conducted in October-November when NPP was at a low. In June, however, NPP values for the GB region exceeded those for the western Mediterranean. Thus, at least part of the divergence between seafloor POC flux and pigment concentrations can be attributed to the usage of annual composite POC flux data, which do not take into account seasonal heterogeneity. However, the difference in pigment concentrations between A1200 and AP3000, both sampled in June, cannot be attributed to seasonal sampling differences. The smaller pigment pool at A1200 may be the consequence of the more extensive, total (i.e. mega-, macro-, and meiofauna, as well as prokaryotes) benthic community at this shallower depth [19], [20]. As for the seamount station, the discrepancy between POC deposition and pigment concentrations may also be a result of the usage of annual composite POC flux in combination with pigment data obtained in October. Nonetheless, an additional explanation for the low pigment concentrations at GB1200 is the strong hydrodynamic forcing, sweeping away fine phytodetrital matter (see earlier).

In contrast to Relexans et al. [73], Gambi and Danovaro [72] and Lampadariou et al. [28], we did not observe lower sediment TOC, TN or TOM values at the less productive stations. Bulk concentrations of these elements do not necessarily represent food availability, since part of nitrogen or carbon containing material might be either intrinsically refractory or unavailable for consumption owing to physical protection by organic or inorganic matrices [74]. Hence, we consider TOC, TN, TOM and C:N to be potential but not definite nematode food indicators. In addition, although these variables are often regarded as a measure of POC deposition, they can be more closely associated with grain size (owing to its relationship with surface: volume ratios) than with OM delivery [75], [76]. In the present study, however, we did not find statistically significant relations between sediment grain size and TN, TOC or TOM.

### Benthic-pelagic Coupling: Effects on Nematode Standing Stock

The present study supports the general notion that deep-sea sediments underlying productive waters harbor elevated meiofaunal (nematode) standing stock [28], [46], [72], [73], [77]–[79]. Also the deep-sea megabenthos attains higher biomass in the more productive western Mediterranean compared to the more oligotrophic eastern basin [80].

The correlation analysis pointed towards the significance of food availability, in terms of POC sedimentation rates and even more importantly sedimentary TN and TOC levels, for nematode standing stock. However, the results obtained for the seamount station indicate that food may not be the only determinant of nematode biomass. Correspondingly, in his review on meiofauna along continental margins, Soltwedel [25] noticed considerable variation in the relationship between CPE and meiofaunal abundances between geographic regions, which he attributed to the interference of other environmental and/or biotic factors. The unusually low standing stock at GB1200 was believed to be the result of the strong hydrodynamical forcing (inferred from the coarse and well-sorted sediments [37], [65]) either through the exertion of physical stress or through the low food availability that comes with it, or both. Food shortage at GB1200 is suggested by the low amounts of phytopigments, nitrogen and organic matter within the sediments. In support, Thistle and Levin [81] documented reduced nematode abundances under experimental strong near-bottom flow.

Opposed to Danovaro et al. [82], the longitudinal trophic gradient was accompanied by a significant drop in individual nematode biomass. Based on an extensive literature analysis, Udalov et al. [83] described a positive effect of grain size and food availability on individual nematode biomass. In contrast, Soetaert et al. [84], who analyzed raw biomass data, found no effect of grain size on individual biomass. In our study area, individual nematode biomass decreased with increasing sedimentary mud content, but strangely also with sedimentary TN concentrations. This finding suggests that bulk TN concentrations might not represent a suitable proxy for the amount of food available to nematodes. Note that the lack of a relationship between nematode size and labile phytodetritus, a potentially better measure for food availability than TN, may be the result of the fact that we had to average environmental and faunal variables per station, thereby expunging (co)variation in both parameters at a smaller spatial scale.

### Benthic-pelagic Coupling: Effects on Nematode Community Structure

There was a highly significant and strong reduction in core surface area from the GB region to the eastern Mediterranean (Spearman rank, r = -0.74, P<0.001). Thus, since sample volume might have an impact on genus composition (smaller samples might contain comparatively less rare genera than larger sediment samples), the observed longitudinal gradient could result partly from the heterogeneity in core surface area. Nevertheless, the generic composition of the nematode assemblages changed gradually from the GB region to the eastern Mediterranean together with several benthic environmental variables (TN, TOC and TOM). The significance of (potential) food availability to the structure of nematode assemblages was also demonstrated by Ingels et al. [85] (TN, chl-a, chl-a:TOC) and Fonseca and Soltwedel [86] (particulate proteins and phospholipids). Similar to Fonseca and Soltwedel [86] (who studied nematode species composition in the Arctic), we noted increased variability in nematode community structure among replicate samples in sediments with reduced phytopigment concentrations. Fonseca and Soltwedel [86] invoked the energy-richness hypothesis [87] as an explanation for this pattern. According to this hypothesis, low energy levels result in small population sizes of species, and local stochastic events restrict species’ distribution ranges.

The gradual change in nematode trophic structure from west to east was mainly driven by the increased relative abundance of predatory/scavenging nematodes (mainly *Sphaerolaimus* and *Metasphaerolaimus*) with longitude. Although they found no statistically significant relationship between predator/scavenger abundance and longitude, Danovaro et al. [82] also noticed a higher representation of this particular feeding guild in the eastern compared to the western Mediterranean. The lower fraction of predatory/scavenging nematodes in the more productive western part of our transect implies that members of this feeding guild do not relate directly to the supply of surface-derived OM. As Gage [2] stated, in oligotrophic regions, organisms feeding upon sedimented POC may suffer a disadvantage compared to those that do not. In support, Sibuet et al. [88] counted most necrophagous amphipods at the most oligotrophic site in the tropical Atlantic. In contrast, sediments from the Nazaré canyon [89] and from several Mediterranean canyons [90], which receive high POC loadings, harbored a higher percentage of predators/scavengers relative to adjacent open slope stations. This paradox calls for more detailed investigations into the environmental drivers of predator/scavenger abundances.

Epistrate feeders were especially abundant at the GB seamount, as observed for Maud Rise in the Antarctic [91] and for sediments surrounding the Paluniro seamount in the western Mediterranean [92]. However, at the Marsili seamount in the western Mediterranean the share of epistrate feeders was limited and as such the dominance of this feeding type in seamount sediments cannot be generalized. Nevertheless, there were very few seamount studies addressing nematode community structure with which we could compare our results, and much more research in this field is definitely needed.

### Conclusions

Along the longitudinal transect from the Galicia Bank region to the eastern Mediterranean, downward benthic-pelagic coupling was evident in terms of phytopigment concentrations, and in standing stock, size, genus and trophic composition of nematodes in bathyal and abyssal sediments. Standing stock seemed to be regulated by POC deposition and benthic potential food indicators (i.e. percentage of nitrogen, organic carbon, and total organic matter), whereas genus composition was only related to the latter. The significant relationship between nematode parameters and POC flux does not necessarily imply these organisms feed upon the sedimented OM directly; for instance bacteria, another potential nematode food source, are often associated with phytodetritus [93].

Climate change is expected to modify the biogeochemical fluxes to the deep sea, which regulate the community structure and function of deep-sea benthic communities [94]. Long-term studies in the northeast Pacific and at the Porcupine abyssal plain have revealed climate-driven variation in the community structure of foraminiferans, mega- and macrofauna in abyssal sediments [95]. The coupling between bathyal and abyssal nematode assemblages and surface water processes, as evidenced in the present study, suggests that it is likely that climate change will affect the composition and function of deep-sea nematodes as well.

## Supporting Information

Table S1 **Average (SE) relative meiofaunal taxon densities (%) per station.** The number of replicate deployments per station can be found in Table 1.
(DOCX)

## References

[1] Lutz M, Dunbar R, Caldeira K (2002) Regional variability in the vertical flux of particulate organic carbon in the ocean interior. Global Biogeochem Cycles 16: 11–18.

[2] Gage JD (2003) Food inputs, utilization, carbon flow and enegetics. In: Tyler P, editor. Ecosystems of the World. 315–382.

[3] De La Rocha CL, Passow U (2007) Factors influencing the sinking of POC and the efficiency of the biological carbon pump. Deep-Sea Res II 54: 639–658.

[4] Gorsky G, Prieur L, Taupier-Letage I, Stemmann L, Picheral M (2002) Large particulate matter in the Western Mediterranean: I. LPM distribution related to mesoscale hydrodynamics. J Mar Syst 33–34: 289–311.

[5] Gorsky G, Borgne RL, Picheral M, Stemmann L (2003) Marine snow latitudinal distribution in the equatorial Pacific along 180°. J Geophys Res 108: 8146 doi:10.1029/2001JC001064.

[6] Zúñiga D, Calafat A, Sanchez-Vidal A, Canals M, Price B, et al (2007) Particulate organic carbon budget in the open Algero-Balearic Basin (Western Mediterranean): Assessment from a one-year sediment trap experiment. Deep-Sea Res I 54: 1530–1548.

[7] Pfannkuche O (1993) Benthic response to the sedimentation of particulate organic-matter at the Biotrans station, 47-degrees-N, 20-degrees-W. Deep-Sea Res II 40: 135–149.

[8] Pfannkuche O, Boetius A, Lochte K, Lundgreen U, Thiel H (1999) Responses of deep-sea benthos to sedimentation patterns in the North-East Atlantic in 1992. Deep-Sea Res I 46: 573–596.

[9] Duineveld GCA, Tselepides A, Witbaard R, Bak RPM, Berghuis EM, et al (2000) Benthic-pelagic coupling in the oligotrophic Cretan sea. Prog Oceanogr 46: 457–480.

[10] Galeron J, Sibuet M, Vanreusel A, Mackenzie K, Gooday AJ, et al (2001) Temporal patterns among meiofauna and macrofauna taxa related to changes in sediment geochemistry at an abyssal NE Atlantic site. Prog Oceanogr 50: 303–324.

[11] Witte U, Aberle N, Sand M, Wenzhofer F (2003) Rapid response of a deep-sea benthic community to POM enrichment: an in situ experimental study. Mar Ecol Prog Ser 251: 27–36.

[12] Ingels J, Van den Driessche P, De Mesel I, Vanhove S, Moens T, et al (2010) Preferred use of bacteria over phytoplankton by deep-sea nematodes in polar regions. Mar Ecol Prog Ser 406: 121–133.

[13] Jeffreys RM, Lavaleye MSS, Bergman MJN, Duineveld GCA, Witbaard R (2011) Do abyssal scavengers use phytodetritus as a food resource? Video and biochemical evidence from the Atlantic and Mediterranean. Deep-Sea Res I 58: 415–428.

[14] Aberle N, Witte U (2003) Deep-sea macrofauna exposed to a simulated sedimentation event in the abyssal NE Atlantic: in situ pulse-chase experiments using 13C-labelled phytodetritus. Mar Ecol Prog Ser 251: 37–47.

[15] Witte U, Wenzhofer F, Sommer S, Boetius A, Heinz P, et al (2003) In situ experimental evidence of the fate of a phytodetritus pulse at the abyssal sea floor. Nature 424: 763–766. 12917681 10.1038/nature01799

[16] Gooday AJ, Pfannkuche O, Lambshead PJD (1996) An apparent lack of response by metazoan meiofauna to phytodetritus deposition in the bathyal north-eastern Atlantic. J Mar Biol Ass UK 76: 297–310.

[17] Pfannkuche O, Sommer S, Kahler A (2000) Coupling between phytodetritus deposition and the small-sized benthic biota in the deep Arabian Sea: analyses of biogenic sediment compounds. Deep-Sea Res II 47: 2805–2833.

[18] Witbaard R, Dunieveld GCA, Van der Weele JA, Berghuis EM, Reyss JP (2000) The benthic response to the seasonal deposition of phytopigments at the Porcupine Abyssal Plain in the North East Atlantic. J Sea Res 43: 15–31.

[19] Rex MA, Etter RJ, Morris JS, Crouse J, McClain CR, et al (2006) Global bathymetric patterns of standing stock and body size in the deep-sea benthos. Mar Ecol Prog Ser 317: 1–8.

[20] Wei CL, Rowe GT, Escobar-Briones E, Boetius A, Soltwedel T, et al (2010) Global patterns and predictions of seafloor biomass using random forests. Plos One 5: e15323. 21209928 10.1371/journal.pone.0015323 PMC3012679

[21] Sommer S, Pfannkuche O (2000) Metazoan meiofauna of the deep Arabian Sea: standing stocks, size spectra and regional variability in relation to monsoon induced enhanced sedimentation regimes of particulate organic matter. Deep-Sea Res II 47: 2957–2977.

[22] Mokievskii V, Udalov A, Azovskii A (2007) Quantitative distribution of meiobenthos in deep-water zones of the World Ocean. Oceanology 47: 797–813.

[23] Boon AR, Duineveld GCA (1996) Phytopigments and fatty acids as molecular markers for the quality of near-bottom particulate organic matter in the North Sea. J Sea Res 35: 279–291.

[24] Stephens MP, Kadko DC, Smith CR, Latasa M (1997) Chlorophyll-a and pheopigments as tracers of labile organic carbon at the central equatorial Pacific seafloor. Geochim Cosmochim Acta 61: 4605–4619.

[25] Soltwedel T (2000) Metazoan meiobenthos along continental margins: a review. Prog Oceanogr 46: 59–84.

[26] Neira C, Sellanes J, Levin LA, Arntz WE (2001) Meiofaunal distributions on the Peru margin: relationship to oxygen and organic matter availability. Deep-Sea Res I 48: 2453–2472.

[27] Tselepides A, Lampadariou N, Hatziyanni E (2004) Distribution of meiobentbos at bathyal depths in the Mediterranean Sea. A comparison between sites of contrasting productivity. Sci Mar 68: 39–51.

[28] Lampadariou N, Tselepides A, Hatziyanni E (2009) Deep-sea meiofaunal and foraminiferal communities along a gradient of primary productivity in the eastern Mediterranean Sea. Sci Mar 73: 337–345.

[29] Danovaro R, Croce della R, Eleftheriou A, Fabiano M, Papadopoulou N, et al (1995) Meiofauna of the deep eastern Mediterranean Sea: distribution and abundance in relation to bacterial biomass, organic matter composition and other environmental factors. Prog Oceanogr 36: 329–341.

[30] Shimanaga M, Nomaki H, Suetsugu K, Murayama M, Kitazato H (2007) Standing stock of deep-sea metazoan meiofauna in the Sulu Sea and adjacent areas. Deep-Sea Res II 54: 131–144.

[31] Tietjen JH (1984) Distribution and species diversity of deep-sea nematodes in the Venezuela basin. Deep-Sea Res I 31: 119–132.

[32] Flach E, Muthumbi A, Heip C (2002) Meiofauna and macrofauna community structure in relation to sediment composition at the Iberian margin compared to the Goban Spur (NE Atlantic). Prog Oceanogr 52: 433–457.

[33] Tselepides A, Lampadariou N (2004) Deep-sea meiofaunal community structure in the Eastern Mediterranean: are trenches benthic hotspots? Deep-Sea Res I 51: 833–847.

[34] Soetaert K, Vanaverbeke J, Heip C, Herman PMJ, Middelburg JJ, et al (1997) Nematode distribution in ocean margin sediments of the Goban Spur (northeast Atlantic) in relation to sediment geochemistry. Deep-Sea Res I 44: 1671–1683.

[35] Joint I, Groom SB, Wollast R, Chou L, Tilstone GH, et al (2002) The response of phytoplankton production to periodic upwelling and relaxation events at the Iberian shelf break: estimates by the C-14 method and by satellite remote sensing. J Mar Syst 32: 219–238.

[36] McClain CR, Chao SY, Atkinson LP, Blanton JO, Decastillejo F (1986) Wind-driven upwelling in the vicinity of Cape Finisterre, Spain. Journal of Geophysical Research-Oceans 91: 8470–8486.

[37] Duineveld GCA, Lavaleye MSS, Berghuis EM (2004) Particle flux and food supply to a seamount cold-water coral community (Galicia Bank, NW Spain). Mar Ecol Prog Ser 277: 13–23.

[38] Tyler PA (2003) The peripheral deep seas. In: Tyler PA, editor. Ecosystems of the World. Amsterdam: Elsevier. 261–293.

[39] Danovaro R, Dinet A, Duineveld G, Tselepides A (1999) Benthic response to particulate fluxes in different trophic environments: a comparison between the Gulf of Lions-Catalan Sea (western-Mediterranean) and the Cretan Sea (eastern-Mediterranean). Prog Oceanogr 44: 287–312.

[40] Turley CM, Bianchi M, Christaki U, Conan P, Harris JRW, et al (2000) Relationship between primary producers and bacteria in an oligotrophic sea - the Mediterranean and biogeochemical implications. Mar Ecol Prog Ser 193: 11–18.

[41] D’Ortenzio F, Ribera d’Alcalà M (2009) On the trophic regimes of the Mediterranean Sea: a satellite analysis. Biogeosciences 6: 139–148 doi:10.5194/bg-6-139-2009.

[42] Bergamasco A, Malanotte-Rizzoli P (2010) The circulation of the Mediterranean Sea: a historical review of experimental investigations. Advances in Oceanography and Limnology 1: 11–28.

[43] Estrada M (1996) Primary production in the northwestern Mediterranean. Sci Mar 60: 55–64.

[44] Bosc E, Bricaud A, Antoine D (2004) Seasonal and interannual variability in algal biomass and primary production in the Mediterranean Sea, as derived from 4 years of SeaWiFS observations. Global Biogeochemical Cycles 18: GB1005.

[45] Bett BJ, Vanreusel A, Vincx M, Soltwedel T, Pfannkuche O, et al (1994) Sampler bias in the quantitative study of deep-sea meiobenthos. Mar Ecol Prog Ser 104: 197–203.

[46] Galeron J, Sibuet M, Mahaut ML, Dinet A (2000) Variation in structure and biomass of the benthic communities at three contrasting sites in the tropical Northeast Atlantic. Mar Ecol Prog Ser 197: 121–137.

[47] Wentworth CK (1922) A scale of grade and class terms for clastic sediments. J Geol 30: 377–392.

[48] Lorenzen CJ, Jeffrey SW (1980) Determination of chlorophyll in seawater. Unesco Tech Pap Mar Sci 35: 1–20.

[49] Behrenfeld MJ, Falkowski PG (1997) Photosynthetic rates derived from satellite-based chlorophyll concentration. Limnol Oceanogr 42: 1–20.

[50] Lutz MJ, Caldeira K, Dunbar RB, Behrenfeld MJ (2007) Seasonal rhythms of net primary production and particulate organic carbon flux to depth describe the efficiency of biological pump in the global ocean. Journal of Geophysical Research-Oceans 112. doi:10.1029/2006JC003706.

[51] Heip C, Vincx M, Vranken G (1985) The ecology of marine nematodes. Oceanogr Mar Biol Ann Rev 23: 399–489.

[52] Wieser W (1953) Die Beziehung zwischen Mundhöhlengestalt, Ernährungsweise und Vorkommen bei freilebenden marinen Nematoden. Eine ökologisch-morphologische Studie. Ark Zool 4: 439–483.

[53] Andrassy I (1956) The determination of volume and weight of nematodes. Acta Zool Hung 2: 1–15.

[54] Clarke K, Warwick R (2001) Change in marine communities: an approach to statistical analysis and interpretation. Plymouth Marine Laboratory: PRIMER-E Ltd.

[55] R Core Team (2012) R: A Language and Environment for Statistical Computing. Vienna, Austria: R Foundation for Statistical Computing. Available: http://www.R-project.org/.

[56] Kim S (2011) ppcor: Partial and Semi-partial (Part) correlation.

[57] Clarke K, Gorley R (2006) PRIMER v6: User Manual/tutorial. Plymouth: Primer-E Ltd.

[58] Anderson MJ, Gorley RN, Clarke KR (2008) PERMANOVA+ for PRIMER: guide for software and statistical methods. Plymouth: Primer-E Ltd.

[59] Moutin T, Raimbault P (2002) Primary production, carbon export and nutrients availability in western and eastern Mediterranean Sea in early summer 1996 (MINOS cruise). J Mar Syst 33–34: 273–288.

[60] Claustre H, Morel A, Hooker SB, Babin M, Antoine D, et al (2002) Is desert dust making oligotrophic waters greener? Geophys Res Lett 29: 1469 doi:10.1029/2001GL014056.

[61] Campbell J, Antoine D, Armstrong R, Arrigo K, Balch W, et al.. (2002) Comparison of algorithms for estimating ocean primary production from surface chlorophyll, temperature, and irradiance. Global Biogeochemical Cycles 16. doi: 10.1029/2001gb001444.

[62] Carr M-E, Friedrichs MAM, Schmeltz M, Noguchi Aita M, Antoine D, et al (2006) A comparison of global estimates of marine primary production from ocean color. Deep-Sea Res II 53: 741–770.

[63] Clark MR, Rowden AA, Schlacher T, Williams A, Consalvey M, et al (2010) The ecology of seamounts: structure, function, and human impacts. Ann Rev Mar Sci 2: 253–278. 10.1146/annurev-marine-120308-081109 21141665

[64] Smith KL, Kaufmann RS, Baldwin RJ, Carlucci AF (2001) Pelagic-benthic coupling in the abyssal eastern North Pacific: An 8-year time-series study of food supply and demand. Limnol Oceanogr 46: 543–556.

[65] Van Weering TCE, De Stigter HC, Boer W, De Haas H (2002) Recent sediment transport and accumulation on the NW Iberian margin. Prog Oceanogr 52: 349–371.

[66] Kerhervé P, Heussner S, Charrière B, Stavrakakis S, Ferrand J-L, et al (1999) Biogeochemistry and dynamics of settling particle fluxes at the Antikythira Strait (Eastern Mediterranean). Prog Oceanogr 44: 651–675.

[67] Gardner WD (2000) Sediment trap sampling in surface waters. In: Hanson RB, Ducklow HW, Field JG, editors. The Changing Ocean Carbon Cycle: A midterm synthesis of the Joint Global Ocean Flux Study. New York: Cambridge University Press. 240–281.

[68] Ducklow H, Steinberg D, Buesseler K (2001) Upper ocean carbon export and the biological pump. Oceanography 14: 50–58.

[69] Legendre L, Rivkin RB (2002) Fluxes of carbon in the upper ocean: regulation by food-web control nodes. Mar Ecol Prog Ser 242: 95–109.

[70] Siokou-Frangou I, Christaki U, Mazzocchi MG, Montresor M, D’ Alcala MR, et al (2010) Plankton in the open Mediterranean Sea: a review. Biogeosciences 7: 1543–1586 doi:10.5194/bg-7-1543-2010.

[71] Pusceddu A, Bianchelli S, Canals M, Sanchez-Vidal A, De Madron XD, et al (2010) Organic matter in sediments of canyons and open slopes of the Portuguese, Catalan, Southern Adriatic and Cretan Sea margins. Deep-Sea Res I 57: 441–457.

[72] Gambi C, Danovaro R (2006) A multiple-scale analysis of metazoan meiofaunal distribution in the deep Mediterranean Sea. Deep-Sea Res I 53: 1117–1134.

[73] Relexans JC, Deming J, Dinet A, Gaillard JF, Sibuet M (1996) Sedimentary organic matter and micro-meiobenthos with relation to trophic conditions in the tropical northeast Atlantic. Deep-Sea Res I 43: 1343–1368.

[74] Burdige DJ (2007) Preservation of organic matter in marine sediments: controls, mechanisms, and an imbalance in sediment organic carbon budgets? Chem Rev 107: 467–485. 17249736 10.1021/cr050347q

[75] Mayer LM (1994) Relationships between mineral surfaces and organic carbon concentrations in soils and sediments. Chem Geol 114: 347–363.

[76] Hedges JI, Keil RG (1995) Sedimentary organic matter preservation: an assessment and speculative synthesis. Mar Chem 49: 81–115.

[77] Lampadariou N, Tselepides A (2006) Spatial variability of meiofaunal communities at areas of contrasting depth and productivity in the Aegean Sea (NE Mediterranean). Prog Oceanogr 69: 19–36.

[78] Bianchelli S, Gambi C, Zeppilli D, Danovaro R (2010) Metazoan meiofauna in deep-sea canyons and adjacent open slopes: A large-scale comparison with focus on the rare taxa. Deep-Sea Res I 57: 420–433.

[79] Gambi C, Lampadariou N, Danovaro R (2010) Latitudinal, longitudinal and bathymetric patterns of abundance, biomass of metazoan meiofauna: importance of the rare taxa and anomalies in the deep Mediterranean Sea. Advances in Oceanography and Limnology 1: 167–197.

[80] Tecchio S, Ramirez-Llodra E, Sarda F (2011) Company JB, Palomera I, et al (2011) Drivers of deep Mediterranean megabenthos communities along longitudinal and bathymetric gradients. Mar Ecol Prog Ser 439: 181–219.

[81] Thistle D, Levin LA (1998) The effect of experimentally increased near-bottom flow on metazoan meiofauna at a deep-sea site, with comparison data on macrofauna. Deep-Sea Res I 45: 625–638.

[82] Danovaro R, Gambi C, Lampadariou N, Tselepides A (2008) Deep-sea nematode biodiversity in the Mediterranean basin: testing for longitudinal, bathymetric and energetic gradients. Ecography 31: 231–244.

[83] Udalov AA, Azovsky AI, Mokievsky VO (2005) Depth-related pattern in nematode size: What does the depth itself really mean? Prog Oceanogr 67: 1–23.

[84] Soetaert K, Franco M, Lampadariou N, Muthumbi A, Steyaert M, et al (2009) Factors affecting nematode biomass, length and width from the shelf to the deep sea. Mar Ecol Prog Ser 392: 123–132.

[85] Ingels J, Tchesunov AV, Vanreusel A (2011) Meiofauna in the Gollum Channels and the Whittard Canyon, Celtic Margin-How Local Environmental Conditions Shape Nematode Structure and Function. Plos One 6: e20094 doi:10.1371/journal.pone.0020094. 21629829 10.1371/journal.pone.0020094 PMC3097227

[86] Fonseca G, Soltwedel T (2009) Regional patterns of nematode assemblages in the Arctic deep seas. Pol Biol 32: 1345–1357.

[87] Hawkins BA, Field R, Cornell HV, Currie DJ, Guégan J-F, et al (2003) Energy, water and broad-scale geographic patterns of species richness. Ecology 84: 3105–3117.

[88] Sibuet M, Albert Charmasson, Deming J, Dinet A, et al (1993) The benthic ecosystem in the 3 EUMELI sites in the northeast tropical Atlantic - general perspectives and initial results on biological abundance and activities. Annales de l’institut océanographique 69: 21–30.

[89] Ingels J, Kiriakoulakis K, Wolff GA, Vanreusel A (2009) Nematode diversity and its relation to the quantity and quality of sedimentary organic matter in the deep Nazaré Canyon, Western Iberian Margin. Deep-Sea Res I 56: 1521–1535.

[90] Soetaert K, Heip C (1995) Nematode assemblages of deep-sea and shelf break sites in the North Atlantic and Mediterranean Sea. Mar Ecol Prog Ser 125: 171–183.

[91] Guilini K, Veit-Köhler G, Mayr C, De Troch M, Van Gansbeke D, et al. (in press) Latitudinal and temporal variability in the community structure and fatty acid composition of deep-sea nematodes in the Southern Ocean. Prog Oceanog.

[92] Pusceddu A, Gambi C, Zeppilli D, Bianchelli S, Danovaro R (2009) Organic matter composition, metazoan meiofauna and nematode biodiversity in Mediterranean deep-sea sediments. Deep-Sea Res II 56: 755–762.

[93] Danovaro R, Marrale D, Della Croce N, Parodi P, Fabiano M (1999) Biochemical composition of sedimentary organic matter and bacterial distribution in the Aegean Sea: trophic state and pelagic-benthic coupling. J Sea Res 42: 117–129.

[94] Smith CR, De Leo FC, Bernardino AF, Sweetman AK, Arbizu PM (2008) Abyssal food limitation, ecosystem structure and climate change. Trends Ecol Evol 23: 518–528. 18584909 10.1016/j.tree.2008.05.002

[95] Smith KL, Ruhl HA, Bett BJ, Billett DSM, Lampitt RS, et al (2009) Climate, carbon cycling, and deep-ocean ecosystems. Proc Natl Acad Sci U S A 106: 19211–19218. 19901326 10.1073/pnas.0908322106 PMC2780780

