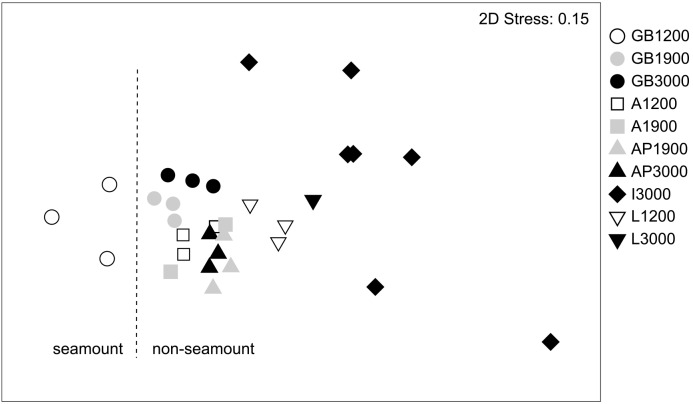# Correction: Benthic-Pelagic Coupling: Effects on Nematode Communities along Southern European Continental Margins

**DOI:** 10.1371/annotation/0db9ed4c-e8be-40cd-a04d-6d0d7928642d

**Published:** 2014-01-08

**Authors:** Ellen Pape, Daniel O. B. Jones, Elena Manini, Tania Nara Bezerra, Ann Vanreusel

In Figure 5 the label 'slope' should read 'non-seamount.'

Please see the correct Figure 5 here: 

**Figure:**