# Supplementary material for: Benthic-Pelagic Coupling: Effects on Nematode Communities along Southern European Continental Margins
Source: PLoS One. 2013 Apr 2;8(4):e59954. doi: 10.1371/journal.pone.0059954 (PMC3615007; doi:10.1371/journal.pone.0059954)
Supplement: Table S1 — Average (SE) relative meiofaunal taxon densities (%) per station. The number of replicate deployments per station can be found in Table 1. (DOCX) [file pone.0059954.s001.docx]

**Table S1.** Average (SE) relative meiofaunal taxon densities (%) per station.

|  | **GB1200** | **GB1900** | **GB3000** | **A1200** | **A1900** | **AP1900** | **AP3000** | **I3000** | **L1200** | **L3000** |
| --- | --- | --- | --- | --- | --- | --- | --- | --- | --- | --- |
| **Amphipoda** | 0.08 (0.08) | - | - | - | - | - | - | - | - | - |
| **Copepoda** |  |  |  |  |  |  |  |  |  |  |
| **adults** | 2.98 (1.49) | 3.11 (0.44) | 3.69 (0.49) | 3.36 (0.51) | 2.20 (0.39) | 6.03 (0.91) | 4.79 (0.14) | 6.43 (2.55) | 2.95 (1.48) | 1.37 |
| **nauplii** | 4.32 (1.04) | 3.35 (0.38) | 5.12 (0.11) | 3.74 (0.62) | 2.85 (1.20) | 4.49 (1.16) | 5.82 (0.63) | 6.44 (1.80) | 3.53 (1.88) | 4.11 |
| **Cumacea** | - | - | 0.03 (0.03) | - | - | - | - | - | - | - |
| **Gnathostimulida** | - | 0.02 (0.02) | - | - | - | - | - | - | - | - |
| **Halacaroidea** | - | 0.04 (0.04) | - | - | - | - | 0.06 (0.06) | - | - | - |
| **Holothuroidea** | - | - | - | - | - | - | - | - | 0.60 (0.60) | - |
| **Isopoda** | 0.04 (0.04) | 0.07 (0.07) | 0.27 (0.21) | - | - | - | - | - | - | - |
| **Kinorhyncha** | - | 0.18 (0.10) | 0.18 (0.06) | 0.29 (0.14) | 0.04 (0.04) | 0.29 (0.04) | - | 0.71 (0.71) | 0.69 (0.43) | - |
| **Nematoda** | 90.69 (2.67) | 91.09 (0.72) | 89.86 (0.76) | 91.08 (1.26) | 91.13 (3.87) | 86.81 (1.57) | 87.96 (0.65) | 83.85 (3.18) | 89.72 (1.95) | 91.78 |
| **Oligochaeta** | - | 0.02 (0.02) | - | - | - | - | - | - | - | - |
| **Ostracoda** | 0.21 (0.11) | 0.19 (0.08) | 0.23 (0.09) | - | 0.04 (0.04) | - | - | - | - | - |
| **Polychaeta** | 1.02 (0.24) | 1.03 (0.07) | 0.60 (0.19) | 0.15 (0.02) | 0.39 (0.05) | 1.12 (0.26) | 0.46 (0.16) | - | 1.55 (0.56) | 1.37 |
| **Rotifera** | 0.15 (0.15) | 0.23 (0.08) | 0.03 (0.03) | 0.49 (0.28) | 2.39 (2.34) | 0.12 (0.06) | 0.19 (0.01) | 1.04 (0.68) | 0.47 (0.28) | - |
| **Tanaidacea** | 0.03 (0.03) | 0.04 (0.02) | - | - | - | - | - | - | - | - |
| **Tardigrada** | 0.48 (0.12) | 0.64 (0.13) | - | 0.88 (0.17) | 0.95 (0.09) | 1.31 (0.38) | 0.71 (0.05) | 1.53 (1.14) | 0.16 (0.16) | 1.37 |
| **Turbellaria** | - | - | - | - | - | - | - | - | 0.33 (0.16) | - |

The number of replicate deployments per station can be found in Table 1.
